# Assessing E-Health adoption readiness using diffusion of innovation theory and the role mediated by each adopter's category in a Mauritian context

**DOI:** 10.1093/inthealth/ihab035

**Published:** 2021-06-10

**Authors:** Manish Putteeraj, Nandhini Bhungee, Jhoti Somanah, Numrata Moty

**Affiliations:** School of Health Sciences, University of Technology Mauritius , 11134, Port Louis, Mauritius; Cardiac Center, Sir Seewoosagur Ramgoolam National (SSRN) Hospital, 21017, Pamplemousses, Mauritius; School of Health Sciences, University of Technology Mauritius , 11134, Port Louis, Mauritius; Faculty of Law, University of Mauritius, 80837, Reduit, Mauritius

**Keywords:** adoption, barriers, diffusion of innovation, E-Health, healthcare

## Abstract

**Background:**

The preparedness of healthcare institutes for the foreseen changes expected to arise through the implementation of E-Health is a significant turning point in determining its success. This should be evaluated through the awareness and readiness of healthcare workers to adopt E-Health technology to reduce health information technology failures.

**Methods:**

This study investigated the relationship between the perceived attributes of innovation and E-Health adoption decisions of healthcare workers as part of a preimplementation process. Using a cross-sectional quantitative approach, the dimensions of the diffusion of innovation (DOI) theory were used to assess the E-Health readiness of 110 healthcare workers in a Mauritian specialized hospital.

**Results:**

A strong inclination towards E-Health adoption was observed, where the prime stimulators were perceived as modernization of healthcare management (84.1%, ẋ=4.19), increased work efficiency through reduction of duplication (77.6%, ẋ=4.10) and faster generation of results (71.1%, ẋ=4.07). The findings of this study also validated the use of five DOI dimensions (i.e. relative advantage, compatibility, complexity, trialability and observability) in a predictability model (F(5, 101)=17.067, p<0.001) towards E-Health adoption. A significant association between ‘adopter category’ and ‘willingness to recommend E-Health adoption’ (χ^2^(8)=74.89, p<0.001) endorsed the fact that physicians and nursing managers have central roles within a social ecosystem to facilitate the diffusion of technology and influence the adoption of innovation.

**Conclusion:**

This is the first study of its kind in Mauritius to successfully characterize each adopter's profile and demonstrate the applicability of the DOI framework to predict the diffusion rate of E-Health platforms, while also highlighting the importance of identifying key opinion leaders who can be primed by innovators regarding the benefits of E-Health platforms, thus ensuring non-disruptive evolutionary innovation in the Mauritian healthcare sector.

## Introduction

Information and communication technology (ICT) is gaining ground in almost every discipline and profession; and the healthcare industry has not been excluded. Electronic communication is critical for the successful growth of most sectors, including healthcare.^1^ To sustain a high-quality healthcare system, it is important to be able to manage healthcare, the key being an ability to access and use appropriate data or information.^2^ E-Health allows for better access to data and information, which in turn may increase the quality of healthcare delivery. E-Health has been an umbrella portraying the joint utilization of electronic correspondence and data, innovation in the healthcare sector, as well as the use of digital data transmitted, stored and recovered electronically for clinical, educational and administrative purposes, both at the local site and remotely.

Over the past few decades, the field of E-Health technology has witnessed significant global development and expansion with the emergence of electronic records systems to more complex technologies in the form of health recommender systems that are superimposed on artificial neural network interfaces.^3–^^5^ Countries such as Germany^6^ and the UK as well as Canada have successfully implemented E-Health platforms, ranging from health information systems to public health-oriented services such as the web-assisted tobacco intervention programme.^7^ Developing countries have not been falling behind, with the implementation of e-pharmacy projects in Malaysia,^8^ telemedicine networks in Bangladesh^9^ and web-based communication tools addressing maternal and child deaths in Peru.^10^ The African continent has not been alienated by such technologies and has shown great interest in exploring and exploiting those avenues to enhance the quality of medical healthcare services. E-Health projects that have gained momentum and public adoption include low-cost sustainable electronic medical records for HIV/AIDS patients in Kenya,^11^ as well as mobile phone-assisted pregnancy support and childcare in Nigeria, Zambia and Malawi among other sub-Saharan African countries.^12^

E-Health is increasingly being employed in combination with tools that build capacity and address the quality of care to improve health systems, use resources efficiently and plan for the progressive adoption of universal health coverage. E-Health enables practitioners to offer services beyond their physical reach. ICT tools such as decision support systems and networks can enable less experienced practitioners to remotely access expert help to make better informed decisions.^13^ E-Health makes health information available to healthcare consumers and improves treatment compliance with regular follow-up and appointments.^14^ The benefits of E-Health also include a shift in focus from individual record-based systems to descriptive data about the population’s health status by enabling access to real-time health-related information, facilitating epidemiological surveys, e-prescription-enhanced systems and the maintenance of hospital services.^15^ This digital medium further enhances consumer support through the reduction of duplicate information at different medical access points and ease of information recall related to case history, and reduces prescription errors through the use of contraindicated medication alerts.^16,17^ E-Health platforms have successfully removed transboundary barriers by creating further accessibility to documents, protocols and real-time monitoring of health dimensions through global exchange of information.^18–^^20^ This utilitarian feature of E-Health has been found to be common across low- and middle-income countries with 31–42% of E-Health processes used for improving communication and geographical access.^21^

As with the various components required for the successful endorsement and implementation of E-Health technologies, a number of barriers exist that impede the development and progression of such media in the healthcare industry. Confidentiality and security issues with digital information have been a limiting factor for a number of projects in developing countries, whereby one of the requirements of patients engaged in the utilization of E-Health systems is the safe management and transfer of data within a virtual network of healthcare service providers.^22^ As discussed by Ahmed et al.,^23^ the emergence of technology as a facilitator in healthcare has also brought along a number of regulatory issues with respect to data protection. The outsourcing of platform development and management, interlaced with the reliance on technology brokers, has raised a number of red flags to ensure the infusion of ethical parameters within their frameworks. While a number of countries have been working towards the inclusion of E-Health policies as part of an E-governance strategy and nationalization of the E-Health network with the likes of the European Patient Smart Open Services project,^24^ countries such as Brazil still require legislative amendments to fully exploit the E-Health sector.^25^ Other prominent barriers to the establishment of effective E-Health platforms include technological readiness with an adequate framework and resources to scale up projects while mitigating disruption to service provision^26^ and increasing productivity in healthcare settings supported by embedding quality assurance frameworks in digital platforms.^27^

A core component in bridging the gap between E-Health policies and consumers, in an effort to increase public acceptance, is the frontline of the industry (i.e. the healthcare service providers). Wickramasinghe et al.^14^ conceded that healthcare providers are the key driving force in pushing E-Health initiatives. Indeed, medical staff, including doctors, nurses and administrative personnel, are the point of contact within a clinical set-up and are pivotal in building trust within an evolving framework. Contrasting findings have been generated with reference to the acceptability and endorsement of such technological changes. Organizational complexities ranging from distribution of workload, reward mechanisms to promote a shift towards utilization of digital platforms and staff training are the most cited barriers at this level.^28,29^ A change in routine with less time spent per patient consultation coupled with the absence of data harmonization across clinical set-ups are also justifications cited for the reluctant transition towards E-Health platforms.^30–^^32^ Patient-practitioner rapport is elemental to increase patients’ adherence to treatment and timely clinical visits, variables which can be positively stimulated through E-Health systems.^33,34^ Contrastingly, empowerment and collaborative frameworks, taking into consideration the constraints of healthcare practitioners while developing and implementing E-Health systems, can potentially facilitate technology inclusion in the healthcare sector.^35^ Numerous studies have also used diffusion of innovation (DOI) theory to understand the push and pull factors stemming from the human capital mediating the successful integration of technology in the healthcare sector, as reviewed by Ward,^36^ Price and St. John^37^ and Greilich et al.^38^

Human capital, a prime feature in innovation adoption, has always been the center of DOI given the prospects of certainty and incertitude while evaluating components of innovation. The innovation decision process, defined as ‘an uncertainty reduction process’, proceeds through sequential steps to gather knowledge, develop attitudes towards innovation mediated by persuasion and choosing to adopt or reject the innovative measure, which leads to the potential implementation and postimplementation evaluation process.^39^ This further anchors the attributes of innovation, that is, relative advantage, compatibility, complexity, trialability and observability, as being critical components for evaluation through the lens of consumers to modulate the rate of adoption.^40^ The adoption of technology in the healthcare sector relies heavily on human factors,^36^ making DOI theory an ideal assessment tool given its positing statement highlighting the influence of an individual's response to new ideas on the rate of diffusion, especially through social grouping.^41^ In addition to aspects of social contagion driving DOI, decision-making mechanisms within innovation processes are also dependent on humans, which in certain cases also include gender patterns.^42,43^

Innovation adoption does not occur simultaneously for all the involved stakeholders and therefore understanding each adopter’s profile and how they motivate change across the organization is essential to harmonize adoption. Characterization of adoption level has been extensively examined by Rogers,^39^ providing the features of each adopter across the spectrum ranging from innovators, early adopters, early and late majority to laggards. Differences in adoption rate appear to be stimulated by risk-taking behaviors, such as for those innovators who can work with uncertainty vs laggards, who prefer certainty and success of innovation before adoption.^39^ The spectrum also reflects a top-down approach whereby early adopters embrace change and innovation fairly rapidly, hence their role as opinion leaders catalyzing the adoption process down the ladder to finally reach the skeptics.^44^ Research by Miller and Sim^45^ showed that practitioners who were willing to canvass their ‘less-convinced’ colleagues to use E-Health platforms without straining the resource cost were ‘early-adopters’. Similar adoption trends have been reported across various departments and organizational frameworks in the healthcare sector.^46–^^48^

In the local context, the rate of adoption of E-Health platforms is either low or underutilized, although Mauritius is undergoing a transformative process to revamp its healthcare infrastructure and boost its image as a destination for medical tourism. In 2008, a health information system was introduced in a specialized institution that only catered for medical records. In 2019, the new Ear, Nose, Throat (ENT) hospital was inaugurated featuring a modern technological infrastructure, with the intention to replicate such platforms in other medical institutions. While the desire to enhance healthcare services has been noted in Mauritius, limited research has been carried out to understand the organizational preparedness and readiness to adopt E-Health. This study will evaluate the attributes of E-Health leading to its endorsement using the DOI framework and investigates the relationship between the perceived attributes of innovation and E-Health adoption decisions of healthcare professionals as part of the preimplementation process.

## Methodology

### Study population

A cross-sectional quantitative study was conducted in a specialized hospital located in the northern part of Mauritius. The overall healthcare staff was enumerated across the departments, that is, from wards to operating theatres and the medical records unit, accounting for a total of 110 respondents with the following designations: medical practitioners, nursing personnel, physiotherapists, pharmacy technicians, medical records officers and perfusionists. Given the relatively small population of the institution, all members were prompted to participate in the study, to which 107 responded positively. This particular medical institution was also selected based on its previous exposure to E-Health platforms, given that it served to assess the feasibility and adoption of electronic health records (EHRs) and the integration of a patient manager and appointment-scheduling module, the latter which is still being used at a minimum functionality. Hence, given the previous exposure to such technologies, this location was ideal to test whether people who have used the technology before would still be inclined towards endorsing a work culture change in the foreseeable future.

### Research instrument design and validation

A quantitative approach was used to gather standardized responses for the study sample. The questionnaire was designed taking into consideration the five dimensions of DOI theory and its integration and adaptability in the healthcare sector. Questionnaire items were formulated after an extensive review of literature focusing on determining each adopter’s profile and DOI variables using original study articles and keywords such as ‘diffusion of innovation’, ‘healthcare’, ‘E-Health’ and ‘health information systems’. The qualitative study by Woodward et al.^49^ also provided a template to probe into awareness and perception of E-Health from each worker’s perspective, assisting in question design. The questionnaire was segmented into four components (Table 1). Given that this study did not focus on one specific component of E-Health such as electronic medical records but instead looked at the holistic application of E-Health in a medical institution, this allowed for a flexible interpretation of E-Health across all departments for a greater outreach, as justified by the general awareness questions in section A. Questions related to past exposure to such platforms were not included given that some of the members would have been rotated after pilot testing of the E-Health platform in the institution. Section B focused on the DOI dimensions with respect to E-Health technologies, with each variable measured on a five-point Likert scale (1=strongly disagree to 5=strongly agree), with six items recorded for each construct. Adopter categorization in section C was based on a 10-point continuum, with 1=not in favor of to 10=strongly in favor of E-Health adoption. The five main categories were superimposed on the scale such that representations were defined as follows: 1–2, laggards; 3–4, late majority; 5–6, early majority; 7–8, early adopters; and 9–10, innovators. Each adopter’s profile was further assessed using category-specific statements (laggards: ‘I like to maintain the traditional system and I will only adopt E-Health because the center is making it compulsory’; late majority: ‘I will only adopt E-Health because most of my colleagues have tried and adopted the innovation successfully’; early majority: ‘I will decide to adopt E-Health based on utility and practical benefits of E-Health’; early adopters: ‘I understand the need to change and look to adopt E-Health to achieve a revolutionary breakthrough’; and innovators: ‘I understand the concept of E-Health and I am able to apply complex technical knowledge essential to adopt E-Health’) to consolidate the rating scale. The concept of technology-use referral is widely accepted as a measure of the adopter’s profile, with early majority to innovators acting as opinion leaders and trendsetters to increase adoption rates.^44^ The referral item was added to reinforce the validity of categorization undertaken. Section D dealt with the collection of demographic information to investigate the effect of gender-specific or job-related variables on the DOI constructs, as well as their potential effects on the adopter’s categorization. Hence, common demographic information such as educational level, age and educational level were excluded from this section.

**Table 1.** Modified DOI questionnaire and variables for E-Health adoption profiling

Section	Description	No. of questions
A	Knowledge	3
B	Perceived attributes of E-Health using the DOI dimensions; i.e. relative advantage, compatibility, complexity, trialability and observability	30
C	Adopter's profile and categorization	3
D	Demographics: gender, department, length of service and job profile	4

### Pilot study, instrument reliability and administration

The questionnaire was pretested with 20 health professionals from a different institution (6 medical practitioners, 10 nurses, 1 pharmacist and 3 medical records officers), selected to closely represent the current study population. Clear comprehension of the questions and fluid transition from one section to another were assessed. Two questions from the initial questionnaire were modified as a result of their double-barreled nature and simplified to ease understanding. Although in this particular survey a predefined scale was not used to measure the five dimensions of DOI theory, the Cronbach α reliability test was applied to report consistency within each of the tested attributes. This was also supplemented by construct validity to ensure the proper characterization of each attribute (Table 2).

**Table 2.** Reliability and construct validity for DOI attributes towards E-Health

	Number of items	Cronbach α coefficient	Bartlett test of sphericity χ^2^ statistic	(Validity) p-value
Relative advantage	6	0.918	485.327	<0.001
Compatibility	6	0.918	421.585	<0.001
Complexity	6	0.880	342.540	<0.001
Triability	6	0.794	281.114	<0.001
Observability	6	0.884	373.926	<0.001

Substitute KMO with Kaiser-Meyer-Olkin (KMO) statistics: 0.819, 0.906, 0.825, 0.750 and 0.822, respectively.

#### Survey administration

Participants were approached in their respective wards after approval from the hospital director and the scope of the study was explained to them. Voluntary participation, with the ability to leave the study at any given point, was mandated. Participants were also briefed on the confidential use and storage of the data collected (i.e. allocated for research purposes only). The questionnaire was self-administered and the study was conducted over a period of 2 mo given the shift schedules of the respondents.

### Hypothesis testing and data analysis

For the purposes of this study, descriptive and inferential data were analyzed using IBM SPSS® Statistics V.20 (IBM Corporation, New York, USA) and Microsoft Excel 2016 (Microsoft Corporation, Washington, USA) was used for graphical representation. Dichotomous and nominal polytomous questions were assessed descriptively, while the five-point Likert items were represented by the method of weighted means. Inferential analysis was conducted by way of correlation, multiple regression and cross-sectional analyses depending on the exploratory nature of the hypothesis set. Normality of data was assessed using the Shapiro-Wilk test to pair parametric or non-parametric tests with the related distribution. A cut-off p-value of 0.05 was taken as the significance level. The three main hypotheses set for this study were as follows:

**H1:** The DOI constructs can be used as a model to predict adoption of E-Health.

A multiple linear regression test was undertaken to determine the predictive ability of the five constructs related to DOI theory used as independent variables (i.e. relative advantage, compatibility, complexity, trialability and observability), where individual computed scores were used against the adoption level as rated. Variation and the independent effect of each construct provided further information on the major dimension of DOI theory, strengthening the prediction model.

**H2:** Adoption of E-Health is influenced by the demographic profile of the adopters.

The demographic items (i.e. gender and duration of service in the medical profession) were of interest as independent variables given their potential influence on E-Health adoption. Given the deviation from normality (W=0.864, p< 0.001), Mann–Whitney U and Kruskal–Wallis H tests, respectively, were used to determine their effect on adoption tendency. Other variables, such as work profile and department, were also considered in this case.

**H3:** Validation of adopters’ categories and their association with DOI constructs as a causality to E-Health adoption.

The categorization of adopters initially obtained through descriptive means was reinforced with pairwise comparisons of the ratings provided by each category of adopters with a focus on laggards. This was supported by testing the E-Health adoption level using the Kruskal–Wallis H test on each adopter’s category to substantiate the willingness to endorse new technologies. Upon validation of the adopter’s profile, computed scores for individual DOI constructs as the dependent variable were tested against the adopter’s category. The Kruskal–Wallis H test was used for all constructs, except for complexity, where one-way ANOVA was used given the normality of distribution (W=0.977, p>0.05).

## Results

### Demographics and E-Health awareness status

Dominant male participation (57.9%) was recorded for this study. Out of all participants, 16.8% had <5 y of work experience opposed to the majority (83.2%), who reported 5 to >20 y. A distinction between working in the current premises and cumulative work experience in the healthcare sector was not made in this case; 29% of the participants were flagged as general staff, given that they had a wide coverage of the entire center, and this included all occupational categories with the exception of nursing professionals (n=76). Participants were also categorized according to the specific wards or departments of the center (Table 3).

**Table 3.** Demographic characteristics of respondents (n=107)

Variable	Attributes	Frequency	%
Gender	Male	62	57.9
	Female	45	42.1
Work unit	Male ward	12	11.2
	Female ward	13	12.2
	ICU	28	26.1
	Operating theatre	14	13.1
	Outpatient department	1	1.0
	Angio department	8	7.4
	General staff	31	29.0
Job denomination	Nursing professional	76	72.0
	Physiotherapist	2	1.9
	Pharmacy technician	2	1.9
	Medical practitioner	19	16.7
	Medical records officer	6	5.6
	Perfusionist	2	1.9
Years of experience in specific field	<1	3	2.8
	1–5	15	14.0
	6–10	19	17.8
	11–15	18	16.8
	16–20	28	26.2
	>20	24	22.4

Most of the respondents acknowledged having heard about E-Health, of whom a smaller proportion were keen on adopting these technologies (Table 4). Shared experience from other health professionals was the main channel through which exposure was gained (32.6%), while other sources such as web-based information, media and, to a lesser extent, academic research (2%), were also cited. In an effort to understand potential limitations and support the DOI constructs, technological use with respect to computer-friendliness and operationalization was determined through a series of questions. Most respondents were adept at using a computer and running suite-based applications (>85%), while responses were divided with respect to advanced processes such as ‘using online library dialogues’ (68.2%) or ‘installing software packages’ (60.7%), which appeared to be more laborious for some participants.

**Table 4.** Awareness status about E-Health platforms

Frequency		%
No, I have never heard about E-Health	5	4.6
Yes, I have heard of E-Health but never used it before	83	77.6
Yes, I am familiar with the application of E-Health and plan to adopt it	19	17.8
Total	107	100.0

### Perceived attributes of E-Health innovation and adoption level

The five constructs (i.e. relative advantage, compatibility, complexity, trialability and observability) were measured using six tailored items per component and were assessed as weighted means (Table 5). Most respondents were in agreement with the relative advantages and compatibility features, endorsing E-Health strategies in their current workplaces, while a more neutral position was recorded for trialability and complexity of the framework, as represented by the computed means per construct (compatibility=4.1, relative advantage=4.0, observability=3.7, complexity=3.5 and trialability=3.4).

**Table 5.** Assessing readiness to E-Health innovations through the DOI constructs

DOI constructs	SD*	D	N	A	SA	Mean**
Relative advantage						
Endorsement of E-Health will be a modernistic approach for the center	1.9	0.9	16.8	35.5	44.9	4.21
E-Health reduces duplicate and inefficient practices	2.8	1.9	17.8	37.4	40.2	4.10
E-Health allows better and faster handling of investigating results	0.9	1.9	26.2	31.8	39.3	4.07
E-Health improves integration of healthcare services	1.9	1.9	19.6	47.7	29.0	4.00
E-Health provides a more collaborative way for health professionals to deliver healthcare	1.9	6.5	22.4	39.3	29.9	3.89
E-Health decreases the incidence of medical errors with the help of clinical support system	2.8	7.5	32.7	33.6	23.4	3.67
Compatibility						
E-Health saves a lot of time	1.9	7.5	6.5	38.3	45.8	4.19
Description of medicines prescription by staff will be accurate and more easily understood	2.8	2.8	12.1	40.2	42.1	4.16
E-Health enables better monitoring and follow-up of controlled substance prescriptions	3.7	2.8	15.0	38.3	40.2	4.08
E-Health will facilitate the building of a stable communication network that connects all involved stakeholders	3.7	3.7	11.2	43.9	37.4	4.07
E-Health improves the workflow in hospitals	4.7	6.5	11.2	37.4	39.3	4.01
E-Health enhances the work I do	2.8	6.5	24.3	40.2	26.2	3.80
Complexity						
Professional stress from data-handling and network security	2.8	7.5	28.0	38.3	23.4	3.72
Lack of familiarity of patients with E-Health	4.7	10.3	20.6	43.0	21.5	3.66
Lack of uniform standards with the center	3.7	12.1	24.3	39.3	20.6	3.61
Lack of time to acquire knowledge and skills about system	5.6	20.6	25.2	29.0	19.6	3.36
Having to work long hours to meet practice demand	7.5	15.0	31.8	32.7	13.1	3.29
The technology used in transferring records between two systems is difficult to master	9.3	18.7	31.8	22.4	17.8	3.21
Trialability						
I would like to try out E-Health since this will set the mark in terms of innovative technologies	4.7	8.4	10.3	45.8	30.8	3.90
I would be able to experiment E-Health if I am more familiar with information technology	4.7	9.3	7.5	54.2	24.3	3.84
I really won't lose much by trying E-Health application even if I don't like it	7.5	10.3	11.2	54.2	16.8	3.63
Professional development related to implement E- Health strategies is offered, so I can try them before I adopt them	13.1	13.1	25.2	36.4	12.1	3.21
Strategies of E-Health are difficult to try at the center	11.2	27.1	27.1	21.5	13.1	2.98
Opportunities to try E-Health application strategies before I adopt them are available	15.0	19.6	20.6	43.0	1.9	2.97
Observability						
I am more likely to use E-Health because there are other departments that benefit from it	5.6	6.5	15.9	41.1	30.8	3.85
I would have no difficulty to tell health professionals in other health institutions about the benefits of E-Health	5.6	3.7	16.8	50.5	23.4	3.82
There is ample evidence in literature to support the effectiveness of E-Health	3.7	5.6	30.8	38.3	21.5	3.68
Opportunities to observe the efficiency and effectiveness of E-Health are available on the media	2.8	8.4	32.7	37.4	18.7	3.61
I can see the application of E-Health strategies being used for many tasks	9.3	9.3	22.4	33.6	25.2	3.56
I have observed other healthcare professionals’ satisfaction with the application of E-Health	2.8	6.5	46.7	30.8	13.1	3.45

*Data were presented as a percentage of the total number of respondents for each item of the constructs under their respective agreement scale.

**Data for each item were presented on a five-point Likert agreement scale (strongly disagree=1, disagree=2, neither agree nor disagree=3, agree=4, strongly agree=5), with the average computed to provide an overview of the perceived inclination towards each item. The scores for the statements have been arranged in descending order of weighted means.

E-Health adoption preference was measured on a 10-point scale (1=least favored to 10=highly favorable), resulting in 66.3% giving a rating of 8 or higher, suggesting a strong inclination towards E-Health adoption. This was supported by a computed mean of 7.73±2.28, indicating a positive preference.

As mentioned, both relative advantage and compatibility appeared to be the prime stimulators for the adoption of E-Health platforms. Statements revolving around modernization of healthcare (80.4%, ẋ=4.21) and increased work efficiency through reduction of duplicates (77.6%, ẋ=4.10) and faster results generation (71.1%, ẋ=4.07) were the major perceived relative advantages of E-Health innovation. Investigating the need for E-Health adoption, most participants were in agreement of the need for such technology to improve on aspects of time management (84.1%, ẋ=4.19) and to ease information transmission among personnel (82.3%, ẋ=4.16), while also improving tasks such as monitoring controlled substances (78.5%, ẋ=4.08). Respondents were fairly neutral regarding the concepts of complexity and trialability of E-Health platforms, with justifications such as the time required to acquire the skills needed to transition to modern systems (48.6%, ẋ=3.36) and the actual opportunity to try the systems before fully endorsing them (44.9%, ẋ=2.97). Lastly, respondents who agreed on the visibility of E-Health platforms were ready to share their experiences with other E-Health-naïve personnel (71.9%, ẋ=3.85); however, most were neutral on similar positive interactions coming from others (43.9%, ẋ=3.45). All data are presented as a cumulative percentage of agreement (agree and strongly agree) and a computed mean for each respective item.

### Generating a model using DOI dimensions to predict E-Health adoption

The effect of individual dimensions (i.e. relative advantage, compatibility, complexity, trialability and observability) were tested on the adoption level of E-Health platforms. These were measured both in terms of directionality of association as well as prediction strength and variance to support the development of a prediction model using the five DOI constructs. A correlational matrix was drawn to represent the strength of association and directionality on adoption level (Table 6). All constructs showed a significant positive correlation to the adoption of E-Health, with the exception of complexity sharing an inverse relationship (–0.427) with respect to the dependent variable, validating the generation of a regression model.

**Table 6.** Correlation matrix of DOI constructs vs E-Health adoption

Constructs	Mean	SD	(1)	(2)	(3)	(4)	(5)
(1) REL	3.98	0.79	–				
(2) COM1	4.05	0.84	0.791**	–			
(3) COM2	3.48	0.88	–0.224*	–0.094	–		
(4) TRI	3.42	0.71	0.407**	0.514**	0.050	–	
(5) OBS	3.66	0.83	0.745**	0.747**	–0.106	0.550**	–
(6) DEP	7.73	2.28	0.499**	0.476**	–0.427**	0.406**	0.521**

*p<0.05; **p<0.01.

Independent variables: COM1, compatibility; COM2, complexity; DEP, the dependent variable, adoption of E-Health; OBS, observability; REL, relative advantage; TRI, trialability.

A regression analysis undertaken to test the ability of the five constructs in mediating the adoption of E-Health validated the predictability of the model (F(5, 101)=17.067, p< 0.001). The model also revealed a good predictive index (R value=0.677) and justified 45.8% of variation in the adoption decision as being influenced by the interactivity of the five constructs. The model was further investigated with regard to the individual effect of the constructs in influencing the dependent variable, resulting in only two predictors, namely, complexity (β=–0.388, t(101)=–5.073, p<0.001) and trialability (β=0.218, t(101)=2.406, p=0.018) significantly impacting the adoption of E-Health, while the others failed to demonstrate an isolated effect (Table 7). A discrepancy in the complexity effect when treated separately can potentially be explained by the effect of multicollinearity, inducing heightened sensitivity to changes reflected in the model. However, its inverse relationship was validated by the standardized β coefficient and its associated t statistic value. Therefore, the DOI constructs proved to be valuable predictors to the adoption of E-Health when treated in unison.

**Table 7.** Isolated effect of DOI constructs on adoption of E-Health

	Unstandardized coefficients		Standardized coefficients		
Model	β	SE	β	t	p
(Constant)	4.617	1.286		3.591	0.001**
Relative Advantage	0.218	0.389	0.075	0.559	0.577
Compatibility	0.252	0.361	0.093	0.699	0.486
Complexity	1.011	0.199	–0.388	–5.073	0.000**
Trialability	0.695	0.289	0.218	2.406	0.018*
Observability	0.643	0.344	0.234	1.869	0.065

*p<0.05; **p<0.01.

### Characterizing adopters and demographic relationships to E-Health adoption

As most respondents were favorable to the adoption of E-Health systems, categorization of the participants was important to determine if a similar pattern of adoption was observed in the local context with regard to the adopter’s spectrum. The lowest percentage recorded was laggards (9.3%) vs the other categories, that is, late majority (15%), early majority (34.6%), early adopters (25.2%) and innovators (15.9%); stratification followed the bell-shaped distribution, as projected by Kaminski,^44^ in the healthcare sector. The majority of participants were positive about recommending the adoption of E-Health to their colleagues (63.6%) vs a lower percentage (31.8%) who were undecided about such referrals. A cross-tabulation of recommendation against adopter’s category satisfactorily mapped the indecisive participants to the expected profile, that is, laggards as represented by 66.7% who were not sure and 33.3% who would not recommend E-Health adoption (Table 8). This was supported by the significant association depicted between adopter category and willingness to recommend E-Health adoption (χ^2^(8)=74.89, p<0.001). There was a significant effect of the adopter’s category in motivating such adoption, as reported by the Kruskal–Wallis H test (χ^2^(4)=36.24, p<0.001) and demarcated by the post-hoc pairwise comparison between laggards and the upper adopter categories on E-Health adoption. The lower t statistic value recorded between laggards–innovators (–5.77) vs late majority−innovators (−3.43) confirmed the significant gap in adoption level recorded between those pairs while all tested pairs reported significant differences at p<0.001 (Table 9). Hence, the data endorse the far-right spectrum of the adopters as influential in innovation.

**Table 8.** Adopter's category and referral preference cross-tabulation

Adopter's category	Will you recommend your colleagues to adopt E-Health?		
	Yes	Not sure	No
Innovator	100.0%	0.0%	0.0%
Early adopter	92.6%	7.4%	0.0%
Early majority	64.9%	35.1%	0.0%
Late majority	18.8%	81.2%	0.0%
Laggard	0.0%	66.7%	33.3%

**Table 9.** Pairwise comparison of adopter category on E-Health adoption

Sample 1–sample 2	Test statistic	SE	Standard test statistic	p	Adjusted p
Laggards–early majority	−47.028	10.790	−4.359	0.000	0.000***
Laggards–early adopters	−48.780	11.207	−4.353	0.000	0.000***
Laggards–innovators	−69.650	12.065	−5.773	0.000	0.000***
Late majority–innovators	−36.125	10.545	−3.426	0.001	0.006**

***p<0.001; **p<0.01.

Demographic variables were also analyzed as potential mediators of adoption level, with reported insignificant effects of gender (U=–1.78, p=0.075), job denomination (χ^2^(6)=4.18, p=0.523) and length of service (χ^2^(6)=3.83, p=0.574). However, assigned department appeared to significantly affect the adoption level (χ^2^(6)=31.05, p<0.001).

### Linking adopter category and DOI constructs

The last step of the study was to identify the effect of the different adopter categories on the individual DOI constructs to tie up the relevance of categorizing adopters to assess innovation adoption, and support the prediction model using DOI dimensions upon adoption level. The separate constructs were treated as dependent variables against the adopter categories, with the relevant tests applied. A significant effect was mediated by the adopter categories on all the DOI constructs as reported for complexity (F (4102)=5.10, p<0.001), relative advantage (χ^2^(4)=26.02, p<0.001), compatibility (χ^2^(4)=22.35, p<0.001), observability (χ^2^(4)=28.68, p<0.001) and trialability (χ^2^(4)=11.06, p<0.05), with the latter demonstrating a weaker effect compared with the other constructs. Hence, in line with the adopter category-mediated effect on adoption of E-Health systems, a similar influence was recorded with regard to the impact on the DOI dimensions representing innovation adoption.

## Discussion

### Awareness and technological friendliness as a precursor to E-Health adoption

The decision to adopt an innovation is an active and dynamic process that begins when an individual becomes aware of the innovation and understands its modality. Innovation adoption is a graded process starting with knowledge and is linked to absorptive capacity as a push factor.^50^ The present data concur with the innovation decision process, starting with knowledge as posited by Rogers,^39^ given that a majority of the respondents demonstrated a good knowledge of E-Health principles even if they did not have any hands-on exposure to those platforms. In this particular case, awareness of the general benefits of such technology would increase implementation efficiency. This first stage also highlights the importance of ‘how-to’ and ‘principles’ knowledge in promoting the acceptance of the innovation.^51,52^ In this particular sample, principles knowledge, which also conceptualizes prior knowledge, can be considered as the prime mediator of the high awareness rate recorded for E-Health, given the past implementation of the EHR platform as a pilot study in the institution providing an overview of the operationalization of the innovation. This variant of knowledge appears to dominate how-to knowledge, even if the latter is intricately linked to technological friendliness.

In the healthcare context, an ability to navigate using basic technology, and users who are equipped with ICT skills with a general understanding of technological tools, appears to facilitate the adoption of E-Health; these characteristics were observed in both healthcare staff and patients.^53,54^ This has also been demonstrated by Olok et al.,^55^ whereby access to computers and the internet facilitates awareness and knowledge, aligning with our data, where the majority of respondents reported being adapt at using computers and running suite-based applications, a fact which endorses the successful transition through the first stage of the innovation decision process when presented to such an implementation process. However, our data, reflecting a low percentage of participants being aware and wanting to adopt E-Health, contradicts findings by Eley et al.,^56^ who effectively demonstrated the tendency of nurses to adopt E-Health tools as a facilitating platform towards their daily tasks. This particular response should be treated cautiously as it may not reflect a barrier towards adoption, but can instead be interpreted as having some reservations towards access to such technology given the prolonged anticipation of such projects, a variable which was not explored. Alternatively, in view of an actual discrepancy, this could potentially be explained by the discontinuation of a past E-Health platform, which could have altered its perceived benefits and acted as a demotivating factor towards the adoption of a new variant of E-Health. Hence, past experience in the form of exposure to successful or failed E-Health platforms on future platform adoption as well as E-Health literacy should be considered an important part of supporting adoption.

### Examining E-Health readiness and adoption through DOI constructs

Introducing innovative technologies in the healthcare industry requires the interplay of key components ranging from organizational readiness to the technology itself. Therefore, dissecting the technological applications through DOI constructs can reveal major strengths and weaknesses, which can be addressed to increase adoption rate. The high mean score obtained for the constructs, namely, compatibility and relative advantage, can be said to be partly motivated by the high proportion of respondents with adequate ICT skills and E-Health awareness, and further shows that medical staff are open to innovative technologies in healthcare. Indeed, compatibility can effectively decrease uncertainty with respect to innovation and increase adoption, while a perceived advantage offers a predisposition to adoption if it outweighs the burden of the process.^57,58^ These constructs are known to positively reinforce the adoption of E-Health innovation, as demonstrated by Rahimi et al.,^59^ and also signifies that the technology to be adopted needs to align and concomitantly modernize the workflow of the personnel, such as improving the efficiency of current tasks, elimination of redundancies or even optimizing the processing time for the technology to be readily adopted. Incompatibility during innovation adoption can come at a high cost, burdening the healthcare system by redirecting valuable time away from highly skilled personnel, especially nurses, towards the use of E-Health platforms.^60^ Hence, a relative assessment of technological friendliness and evaluation of the training needs for medical personnel is critical to improve adoption through paired compatibility.

Complexity of a technology, relating to the perceived ease of use, can negatively impact innovation adoption among nurses in the healthcare sector.^61^ Our present findings, reporting a similarly significant negative effect of complexity on adoption of E-Health platforms and its impact on relative advantage, suggests that increased intricacy of the technological interface operability would make it appear as less beneficial, given the exhaustive process of navigation and time spent on the system, which will certainly culminate in a decreased adoption rate. Similar observations were reported in Alkhateeb et al.,^62^ whereby user-friendliness acts as a key determinant to potentiate adoption, hence adhering to the principle of simplification when it comes to end users of E-Health technologies.^63^ Furthermore, complexity does not only rely on the usability by healthcare professionals, but also on the general technological framework, including hardware technicalities such as bandwidth, real-time processing and data transfer across platforms, which may demotivate the personnel from adopting the technology if it delays their daily operations.^64^ Therefore, the inclusion of the main users of E-Health platforms during the design phase or more tailored services with respect to the platform interface would potentially ease the implementation and adoption process, rather than imposing a set-up that may not be well received by the end users.

Trialability and observability are dimensions that influence the intent of adoption based on active and passive user experience, respectively. The present data show that healthcare professionals did not fully agree on the potential of experiencing the technology first-hand, therefore expressed skepticism towards trying innovative technologies, which may be associated with the discontinued past pilot E-Health platform. Trialability, referring to the use of the technology before its adoption, is known to mediate the perceived ease of use, as reported by Abdekhoda et al.,^65^ where physicians were exposed to compatibility pairing. Similarly, healthcare workers who were not provided with a trial run of a computerized care plan were not satisfied with its specifications and purpose, decreasing compatibility.^58^ Observability in tandem with trialability can increase adoption.^39^ Past studies revealed that healthcare professionals who were given the opportunity to test and visually observe the outcomes through simulations were more inclined towards innovation adoption.^66^ However, although trialability can have an individual effect on end users, observability as a dimension of innovation adoption is most potent when visibility of the technology is primed through group practice,^62^ corroborating the present response of individuals ready to embark on the adoption process if others derive satisfaction from such platforms, a feature associated with modelling.^67^ Lastly, we also observed the department-mediated effect on adoption, hence plausibly indicating the role of some departments as a catalyst given the higher prevalence of adoption, while also acting as a modelling unit for others to follow. Our findings are in line with past studies that show that enhancing the first-time experience, along with the sharing of successes through trialability from the users, can motivate individuals towards its adoption and, to a certain degree, influence its compatibility.

Inclusion of all the DOI constructs is known to be a potent predictor of the implementation success of an innovation.^67^ A predictive model drawn showed the significant impact of the constructs on the adoption of E-Health platforms, consistent with that reported by Olok et al.^55^ Rogers^39^ posited that these attributes, when considered in relation to an innovation, reduce the uncertainty surrounding it and increase the adoption readiness, in this case, the E-Health platform. From the items characterizing their respective constructs, healthcare professionals perceive technology in their field as a modernistic approach, which can plausibly optimize their workflow, and are motivated to adopt the technology if they have an opportunity to familiarize themselves with a platform with increased visibility in terms of its ease of use and successful implementation. However, increased complexity around technology with regard to data handling, requiring rigorous training, acts as a deterrent to adoption, hence its inverse relationship with the relative advantages of such platforms. Hence, the interactivity between the individual dimensions, as demonstrated by the significantly strong correlation between relative advantage and compatibility or observability vs its negative relationship with complexity, endorses the application of DOI constructs as predictors to measure the diffusion of technology in the Mauritian healthcare sector. Coupling the application of DOI theory in the current study with other frameworks geared towards understanding technology adoption, such as the technology acceptance model (TAM) or unified theory of acceptance and use of technology (UTAUT), would consolidate the findings, given that these models measure variables that focus more on behavioral intents and their precipitating factors in the form of self-efficacy, attitude, expected performance and effort requirements, among others, to assess the successful acceptance and use of technology.^68–70^ This study therefore warrants the generation of a hybrid model, as developed by Karahoca et al.,^71^ to gather a holistic overview of the factors that account for the diffusion capacity of a technology with respect to the end users.

### The importance of mixed adopter category to motivate E-Health adoption

The adopter's profile and spectrum, observed through a bell-shaped distribution denoting an increased presence of early majority adopters, is consistent with Vedel et al.,^72^ highlighting the occurrence of a spectrum to motivate innovation adoption. A particular inconsistency from our study shows a higher percentage of early adopters vs the late majority, which could be justified by past exposure to familiar platforms, priming them to adopt the technology more readily. Furthermore, the fact that a high proportion of respondents were IT-skilled could also potentiate their adoption latency, a variable which is considered to be a driver for adoption.^72^ However, this should be interpreted with caution as technophilic individuals do not necessarily adopt an innovation unless the perceived relative advantage and compatibility are visible, along with other conditions which need to be satisfied.^60^ The hierarchy of innovation adoption in the healthcare industry is particularly important as the innovators who can be considered as the product testers are not impacted by failures, but rather can provide valuable feedback on the technology, and to a certain extent modulate the initial introduction of the technology through dissemination to the early adopters in a healthcare set-up. Within those lines, early adopters are the quintessential links of the chain as they form part of a social ecosystem where their role as opinion leaders motivates the diffusion of the technology across the healthcare set-up, and provide their support of the technology after rigorous trialability accompanied by empirical justifications. This further highlights the importance of exposure/observability when it comes to early adopters and their additional responsibility and accountability to stimulate adoption across the spectrum. In this particular case, opinion leaders would be the physicians and senior nursing professionals tasked with reeling in the early and late majority, as well as the laggards, towards innovation adoption.

Our findings are consistent with Rogers’ theory of differential adoption rate in relation to the adopter's category, while also acknowledging the existing gap at the far end of the spectrum, that is, the laggards vs the innovators or early adopters in terms of readiness towards E-Health adoption. Leadership within healthcare institutions is important in driving the acceptance and adoption of innovation. The roles of physicians and nurse managers are often cited as precipitators of E-Health developments and practices, tasks which are not limited to lateral peer influence, but also to motivate the vertical diffusion of the technology.^73^ They should be further regarded as network brokers given their general status within the healthcare institution and their nodal ability, linking staff and patients to promote the usefulness of technology in healthcare.^74^ The current study, although not segregating the status of nursing professionals and physicians, points to the possible implementation of a top-down approach using those network brokers given the staffing distribution of the healthcare institution. Furthermore, the fact that individuals from the far-left spectrum are more skeptical in nature strengthens the notion of the general pool of adopters driving adoption, even if they tend to tread with more care, as opposed to the innovators. The reluctance of the late majority and laggards in diffusing the technology, as represented in this study by their uncertainty towards referring E-Health platforms to their peers, can potentially be justified by the fact that this conservative cluster may be intolerant towards technological change or belong to a more mature population who represent the skeptics, as shown in previous studies.^75,76^ However, given that those variables were not studied in depth, the conservative attributes of the participants from these categories are extrapolations at this stage.

A modulating effect of the adopter's category on the perception of the DOI constructs was also noted. As with all technology diffusion, E-Health platforms follow a similar pattern of adoption rate, such that there is no exclusive dependence on the adopter's characteristics but rather on the interactivity of the latter and the DOI dimensions. Escobar-Rodríguez and Romero-Alonso^46^ noted the behavioral difference between early and late adopters in their willingness to adopt IT innovations in healthcare institutions. Our data align with such a notion given the general decline in referral tendency as we move away from innovators to laggards, coupled with the observed differences in perceived DOI attributes. Riverola et al.^77^ describe early adopters as originating from a cosmopolite stratum bearing traits of young age, high education and income status compared with the late adopters, explaining the relative indifference towards observability, trialability and compatibility. This would complement the differences observed in DOI constructs, such that the low percentage of late adopters in the form of late majority and laggards would be more concerned with those constructs, and bothered by the complexity and relative advantage of the E-Health platform, which could be depicted as a sign of reluctance to embrace the innovation. This study pairs with the general consensus of exploring the inherent attributes within each adopter category, such as socioeconomic status, to better understand its influence on the individual DOI constructs.^78^ In this case, although a physician or nursing manager viewed as an opinion leader would adopt E-Health innovations faster than the late adopters, this does not necessarily reflect personal innovativeness, as this might arise from a personalization aspect such as maintaining a credible figure rather than overtly accepting innovation.

### Limitations

A major limitation of the current study was the heightened importance given to the DOI dimensions and participants’ perceptions while downplaying the other components of DOI theory. The innovation adoption process should be investigated in future studies given the influence of DOI constructs on individual stages of the adoption process. The potency of the communication channels within participants’ social network would help to understand the role of the different staffing strata, effectiveness of lateral peer influence and vertical information diffusion within the healthcare institution, given the effect of social reinforcement on the rate of diffusion.^79^ These variables could also potentially be mapped against their related adopter's category. In the present study, the level of ICT skills or the allocated time for adequate ICT training generated mixed attitudes with regards to the complexity of the innovation. However, the perceived complexity of previous and future E-Health platforms was not properly characterized given that: (1) the study was not based on post-hoc implementation and discontinuation, (2) actual knowledge about E-Health through more intricate descriptions such as being task-oriented or patient-oriented was omitted and (3) stratification of participants according to past exposure to the E-Health platform was not undertaken. The present study, by focusing primarily on the individuals representing the main actors diffusing innovation, did not cater for external factors such as organizational components as exemplified by staffing structure, capacity building through training and learning climate,^80^ as well as behavioral components in the form of user acceptance, perceived usefulness, self-efficacy through empowerment and personal interests,^62,74^ factors which could be inhibiting in nature. This calls for modified approaches when assessing the diffusion of innovation using hybrid models merging TAM, DOI, UTAUT and technology organization and environment frameworks to integrate most of the variables within an institution to map diffusion.^81,82^ Extrinsic pressure forces such as governmental stewardships enforced through E-Health standards, policies and legislation should also be included to determine whether measures such as incentivization would fasten the diffusion process by reeling in the late adopters and reinforcing positive attitudes towards technological change.

### Future direction

While the present study has focused primarily on the end-user adoption of E-Health technology, an important barrier to the adoption of an industry 5.0 culture in healthcare (i.e. increased hybrid platforms with technology and human capital) lies further upstream with healthcare policies and funding. Good healthcare metrics inclusive of technological endorsements positively affect healthcare expenditures.^83^ Gross domestic product (GDP) growth allows channeling of more funds into the healthcare sector, as observed in Brazil, Russia, India, China and South Africa.^84^ A global analysis of developed and developing nations such as the G7, emerging market 7 and ‘Next Eleven’ countries has effectively demonstrated the need to increase healthcare expenditures to support better outreach and treatment quality, hence, inevitably impacting on the out-of-pocket (OOP) expenditures, which tend to be a major bottleneck in healthcare affordability.^85,86^ Locally, a notable increase in healthcare expenditure per GDP of 4.6% to 5.7% was observed for 2014 to 2017, whereby OOP expenses accounted for 48% of total expenditure compared with domestic government expenditure of 50.8%.^87^ However, a contraction in GDP has been forecasted as an immediate impact of the COVID-19 pandemic, consequentially impacting on government expenditure, as reported by the 8% decrease in budget allocation to the healthcare sector for the 2020–2021 financial year compared with the previous year.^88^ Hence, a technical analysis of the budgetary constraints and their impact on the deployment of modern technologies inclusive of E-Health platforms would consolidate the existing literature on technological adoption and healthcare economics in Mauritius.

### Conclusion

Understanding each adopter's perception towards innovation is elemental in supporting the diffusion of technology. This study has successfully characterized adopters’ profiles in a specific Mauritian healthcare institution while demonstrating the applicability of the DOI framework in predicting the diffusion rate of E-Health platforms. However, the contextual application of innovations with respect to the adopter should also be considered rather than focusing entirely on the platform's performance. Most importantly, with its focus being the status of innovation adoption, the current study further showed the importance of identifying the key opinion leaders in the form of early adopters, who can be primed by the innovators regarding the benefits of E-Health platforms and, in turn, given their fundamental role within their network, facilitate the diffusion of the technology across the other adopters until a point of saturation is reached. This also highlights the central role of physicians and nursing managers within a social ecosystem to facilitate diffusion of technology. Adoption of innovation in the Mauritian healthcare sector is thought to replicate the trend depicted in other countries with an evolving industry. Although findings from this study relate to the postanalysis of past exposure to a discontinued E-Health platform, this may serve as a precursor towards the endogenous and exogenous factors that need to be considered before the diffusion of any E-Health innovation across the country.

## Data Availability

The data that support the findings of this study are available from the corresponding author upon reasonable request.
